# Mapping Magnetic Properties and Relaxation in Vanadium(IV) Complexes with Lanthanides by Electron Paramagnetic Resonance

**DOI:** 10.3390/molecules24244582

**Published:** 2019-12-14

**Authors:** Ivan V. Kurganskii, Evgeniya S. Bazhina, Alexander A. Korlyukov, Konstantin A. Babeshkin, Nikolay N. Efimov, Mikhail A. Kiskin, Sergey L. Veber, Alexey A. Sidorov, Igor L. Eremenko, Matvey V. Fedin

**Affiliations:** 1 International Tomography Center SB RAS, 630090 Novosibirsk, Russia; ivan.kurganskiy@yandex.ru (I.V.K.); sergey.veber@tomo.nsc.ru (S.L.V.); 2 Novosibirsk State University, 630090 Novosibirsk, Russia; 3 N.S. Kurnakov Institute of General and Inorganic Chemistry of the Russian Academy of Science, 119991 Moscow, Russia; evgenia-vo@mail.ru (E.S.B.); bkonstantan@yandex.ru (K.A.B.); nnefimov@yandex.ru (N.N.E.); mkiskin@igic.ras.ru (M.A.K.); sidorov@igic.ras.ru (A.A.S.); ilerem@igic.ras.ru (I.L.E.); 4 Nesmeyanov Institute of Organoelement Compounds of the Russian Academy of Sciences, 119991 Moscow, Russia; alex@xrlab.ineos.ac.ru

**Keywords:** vanadium(IV) ions, EPR, relaxation times

## Abstract

Vanadium(IV) complexes are actively studied as potential candidates for molecular spin qubits operating at room temperatures. They have longer electron spin decoherence times than many other transition ions, being the key property for applications in quantum information processing. In most cases reported to date, the molecular complexes were optimized through the design for this purpose. In this work, we investigate the relaxation properties of vanadium(IV) ions incorporated in complexes with lanthanides using electron paramagnetic resonance (EPR). In all cases, the VO_6_ moieties with no nuclear spins in the first coordination sphere are addressed. We develop and implement the approaches for facile diagnostics of relaxation characteristics in individual VO_6_ moieties of such compounds. Remarkably, the estimated relaxation times are found to be close to those of other vanadium-based qubits obtained previously. In the future, a synergistic combination of qubit-friendly properties of vanadium ions with single-molecule magnetism and luminescence of lanthanides can be pursued to realize new functionalities of such materials.

## 1. Introduction

The search for molecular spin qubits with suitable physical properties is one of the main targets on the way to the implementation of quantum spin technologies and quantum computing [1,2]. Among other candidates for molecular spin qubits, vanadium-based complexes attracted special attention during the past decade due to their long decoherence times, both at cryogenic and room temperatures [3,4,5,6,7,8,9,10,11,12,13,14,15]. The vanadium(IV) ion has an electron spin *S* = 1/2 and a nuclear spin *I* = 7/2 for stable isotope ^51^V of nearly 100% natural abundance. These properties determine the intrinsically longer electron spin relaxation times compared to high-spin (*S* > 1/2) ions, and, at the same time, provide a set of nuclear spin states for manipulation of multiple coherences. Some previous works were concerned with the frozen solutions of vanadium(IV) complexes [3,4,7,13,14], while the others considered solid-state compounds and their relaxation properties. The longest decoherence time (also called dephasing, phase memory, sometimes transverse relaxation time) at room temperature *T*_m_~1 μs was obtained for vanadyl phthalocyanine complexes with nuclear spin-free ligands in the solid-state [6].

Seeking for the molecular systems with the longest decoherence times available, one should also consider some of the stable organic radicals (*S* = 1/2), which could have longer *T*_m_ than vanadium ions at room temperatures. For example, triarylmethyl (trityl) radicals are actively used nowadays in biological applications of EPR at ambient conditions [16,17,18]. For instance, it was demonstrated that even in aqueous solution at 310 K the dephasing time of the (immobilized) trityl label attached to DNA can reach *T*_m_ = 1.4 μs, whereas in glassy trehalose powder at 300 K it is *T*_m_ = 2.2 μs, both being longer than the record value for vanadium-based qubit being ~1 μs at 300 K [5,6]. Therefore, in terms of the relaxation properties, vanadium-based qubits need further development to outperform the existing molecular competitors such as trityls.

Most of the studies on vanadium-based qubit candidates were dealing with specially designed molecular complexes, even if they were later assembled into the dedicated architectures such as MOFs [11]. In this work, we approach the same task from the opposite side. In fact, we selected vanadium-containing complexes of lanthanides (initially designed for another research) and attempted to investigate what would be the intrinsic electron spin relaxation of vanadium moieties in such more complicated compounds. As we show below, such ad-hoc testing yielded unexpectedly promising results, and in this way, it outlines possible future strategies for designing complex structures incorporating vanadium-based qubits.

More specifically, it is often attractive to design compounds with multiple functionalities. For instance, developing complexes, which include lanthanide ions along with vanadium building blocks, would potentially pave the way for combining luminescent characteristics and even single-molecule magnetism with qubit-friendly properties of vanadium ions. Although at the moment, this is feasible only in the long run, investigation of representative compounds of this type might be insightful. With this purpose, we have studied the spectroscopic and relaxation properties in a series of vanadium complexes with lanthanides using EPR spectroscopy. All these complexes include VO_6_ octahedral units, which to the best of our knowledge, have not yet been considered as potential qubits at room temperatures (only low-temperature relaxation studies were performed for some complexes [13,14]). At the first step of this research, diamagnetic lanthanides in the complexes with paramagnetic vanadium ions were addressed. Below we describe the developed approaches, the obtained results, and compare them with those of other previously studied vanadium-based molecular spin qubits.

## 2. Results and Discussion

### 2.1. Synthesis

Compounds {[KY(VO)_2_(cbdc)_4_(H_2_O)_11_]·2H_2_O}_2_ (**I**) and {[KLu(VO)_2_(cbdc)_4_(H_2_O)_11_]·2H_2_O}_2_ (**II**) have been synthesized by the reaction of aqueous vanadyl nitrate [prepared via metathesis between VOSO_4_·3H_2_O and Ba(NO_3_)_2_ in water] with a freshly prepared aqueous solution of K_2_cbdc (cbdc^2–^ is cyclobutane-1,1-dicarboxylate anion) and Ln(NO_3_)_3_ (**I**: Ln = Y, **II**: Ln = Lu) [19]. The replacement of K_2_cbdc by Na_2_cbdc in similar reactions led to the formation of compounds [NaY(VO)_2_(cbdc)_4_(H_2_O)_10_]_n_ (**III**) and [NaLu(VO)_2_(cbdc)_4_(H_2_O)_10_]_n_ (**IV**).

### 2.2. Crystal Structure Description

**Figure 1:**
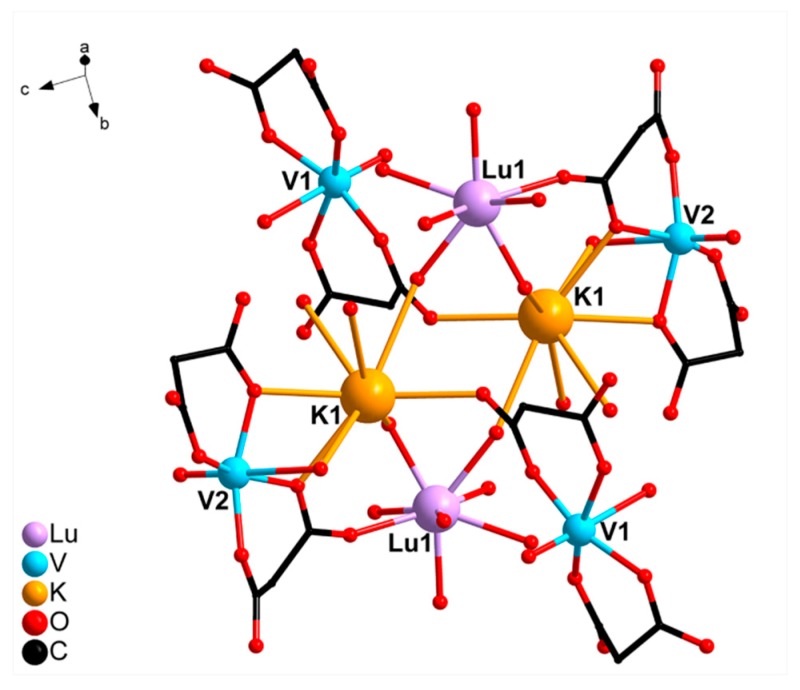
Structure of octanuclear complex **II** (hydrogen atoms and cyclobutane fragments of cbdc are omitted).

The structures of compounds **I** and **II** were described in detail previously [19]. Compounds **I** and **II** are isostructural octanuclear molecular complexes formed by two binuclear cationic fragments [Ln(VO)(cbdc)_2_(H_2_O)_7_]^+^, two anionic moieties [VO(cbdc)_2_(H_2_O)]^2−^, and two K^+^ cations, which connect them. Figure 1 shows the structure of compound **II**, which is similar to the structure of the representative compound **I**.

**Figure 2:**
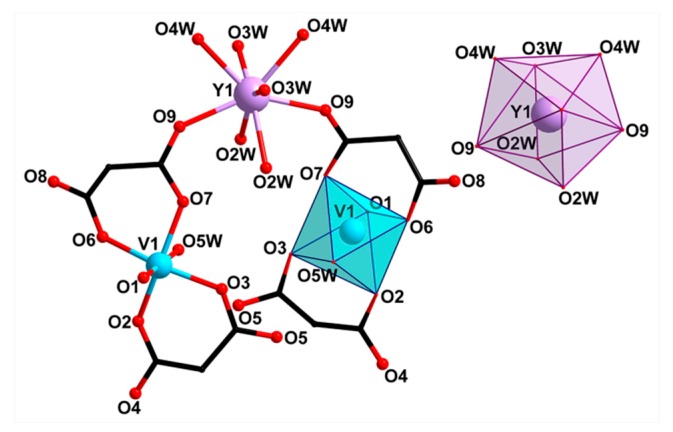
Structure of trinuclear units {YV_2_}^−^ and representation of coordination polyhedra VO_6_ and YO_8_ in **III** (hydrogen atoms and cyclobutane fragments of cbdc are omitted).

**Figure 3:**
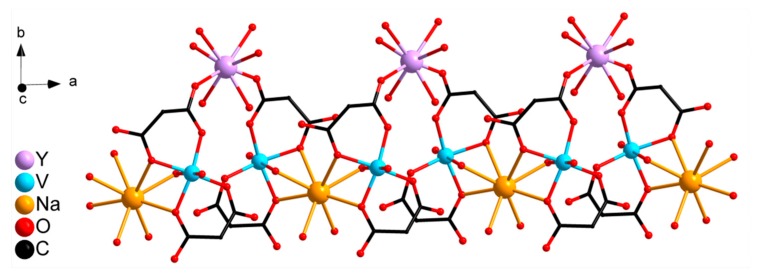
Fragment of polymeric chain **III**: binding of units {YV_2_}^−^ by Na^+^ cations (hydrogen atoms and cyclobutane fragments of cbdc are omitted).

The structure of complex **III** was determined by single-crystal XRD. It comprises two mononuclear bis-chelate dianions [(VO)(cbdc)_2_(H_2_O)]^2−^ containing crystallographically equivalent V1 atoms. The coordination polyhedron of the V1 atom can be described as a distorted octahedron (chromophore VO_6_) formed by four O atoms of two chelating cbdc^2−^ anions in the equatorial plane (V–O 1.97–2.01 Å) and the water O (V–O 2.28 Å) and vanadyl O atoms (V=O 1.60 Å) in the axial positions (Figure 2, selected bond lengths, distances, and angles are given in Table S1). Dianions [(VO)(cbdc)_2_(H_2_O)]^2−^ in **III** are linked to the Y atom via coordination of the carboxylate O atoms of cbdc^2–^ anions, thus forming the anionic complex [Y(VO)_2_(cbdc)_4_(H_2_O)_8_]^−^ ({YV_2_}) having the trinuclear metal core YV_2_ (Figure 2). The distances V···Y and V···V within the trinuclear unit {YV_2_} are equal to 5.69 and 6.35 Å, respectively. The coordination polyhedron of the Y atom (chromophore YO_8_) can be described as a distorted triangular dodecahedron (TDD-8) [20], the vertexes of which are occupied by 6 water oxygen atoms (O2W, O3W, O4W) and 2 carboxylate oxygen atoms (O9) (Y–O 2.33‒2.36 Å) (Figure 2). In the crystals of **III**, the heterometallic units {YV_2_} are linked into a 1D polymeric structure via Na atoms coordinated to the carboxylate O atoms (Na–O 2.63 Å) and water O atoms (Na–O 2.41 Å) of vanadyl-containing moieties {(VO)(cbdc)_2_(H_2_O)} of neighboring {YV_2_} units. In addition, each Na atom coordinates two O atoms of water molecules (Na–O1W 2.44 Å) (Figure 3).

The complexes **III** and **IV** are isostructural according to powder XRD (see Figure S1). Thus, the structure of V-Ln heterometallic compounds with cbdc^2−^ anions is determined by the presence and the nature of alkali cation: complexes **I** and **II** are molecular, and compounds **III** and **IV** are polymeric.

Remarkably, in all cases, the first coordination sphere of vanadium ions contains only 6 oxygens and, therefore, is nuclear spin-free. This is one of the prerequisites for good relaxation properties of these moieties, which will be demonstrated in the sections below.

### 2.3. CW EPR Study of Solid Phases

The temperature dependence of magnetic susceptibility for compounds **I** and **II** was reported and discussed previously [19] (see Figure S2). The vanadium units are well magnetically isolated from each other in **I**, **II**, and magnetic susceptibility is essentially temperature-independent within 2–300 K. The compounds **III** and **IV** feature very similar vanadium-based building blocks, allowing us to assume that all general trends should be the same for compounds **I**–**IV**. Indeed, the observed trends in CW EPR for **I**–**IV** were very similar.

**Figure 4:**
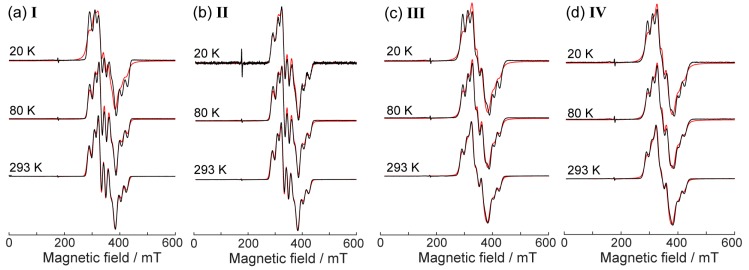
X-band CW EPR spectra of studied compounds (polycrystalline powders) vs. temperature. Experimental spectra are shown in black, simulations in red. (**a**–**d**) Temperatures and compound titles are indicated. The narrow signal at ~175 mT refers to the impurity in the resonator.

Figure 4 shows CW EPR spectra of studied compounds **I**–**IV** vs. temperature at 20–293 K. Only slight variations of the spectral shapes vs. temperature were observed, all being assigned to the changes in the linewidth of the multicomponent spectra described by the same principle spectroscopic parameters (g- and hyperfine interaction (A) tensors). These spectra can be satisfactorily simulated in a model of one V^4+^ ion with the pronounced splitting due to the hyperfine interaction with ^51^V nucleus having *I* = 7/2 spin (see Figure 4 and Table 1 for parameters). No exchange coupling between two vanadium moieties had to be taken into account, which agrees with magnetic susceptibility data. This is reasonable since the two vanadium moieties in the structure of these complexes were sufficiently distant providing weak exchange couplings. Most evidently, in all spectra shown in Figure 4 we do observe hyperfine splitting characteristic for single vanadium(IV) [6], which would not be the case if the intermolecular exchange couplings were sensible. Thus, in principle, the vanadium moieties in compounds **I**–**IV** were well magnetically isolated, and the similarity of the EPR parameters for **I**, **II** vs. **III**, I**V** suggests their similar magnetic behaviors.

### 2.4. Relaxation Properties of Complexes in Frozen Solutions

The key property of vanadium-based potential spin qubits is their long phase memory time (*T*_m_) compared to many other transition ions. This time has to be measured using pulse EPR, most conveniently by two-pulse Hahn echo sequence (π/2 - τ - π - τ - echo), where interpulse delay τ is incremented. However, despite the fact that the vanadium moieties were sufficiently well isolated from each other in the studied compounds (see above), we did not succeed in recording any pulse EPR data on solid-state compounds. This reasonably stems from still high magnetic concentration favoring interspin dipolar couplings and a drastic shortening of *T*_m_. In order to measure the relaxation of individual ions in the solid-state, one has to perform diamagnetic dilution of the crystal, where isostructural diamagnetic analogs have to be introduced in concentrations close to 100%. For instance, this approach was used in previous studies of the relaxation properties of vanadium ions [5].

**Figure 5:**
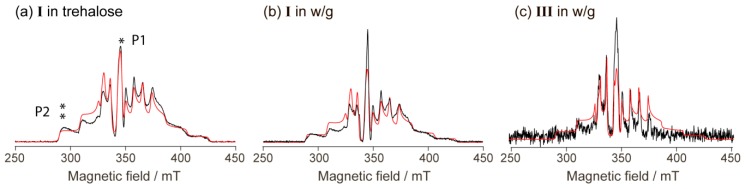
Echo-detected EPR spectra of **I** and **III** (black – experimental, red – simulated). (**a**) **I** in glassy trehalose, (**b**) **I** in the frozen water/glycerol (w/g) solution, (**c**) **III** in the frozen water/glycerol (w/g) solution. T = 80 K. The two spectral positions where the relaxation measurements were done are shown by asterisks (*, **) and P1/P2 signs in (**a**).

In this work, we used a noticeably simpler strategy. We dissolved two representative compounds (**I** and **III**) in glass-forming water/glycerol mixture (~50/50% *v*/*v*) and recorded two-pulse echo-detected (ED) EPR spectra in such frozen (glassy) solutions (Figure 5). Note that mostly vanadium-based candidates for spin qubits were investigated in the solid crystalline state; however, a few earlier works used dissolution as well [3,4,7,13,14]. In particular, this approach allowed obtaining very long decoherence times at low temperatures, especially in nuclear spin-free solvents. In our case, we were most interested in decoherence times at ambient temperatures, therefore, we did not use deuterated solvents whose role is primary only at low temperatures [4].

Remarkably, the ED EPR spectra of **I** and **III** (Figure 5) can be reasonably well simulated using the same g- and A-tensors as the spectra of the solid phases (see Figure 4 and parameters in Table 1), only the linewidth had to be adjusted. The line intensities here could not be matched well, perhaps due to the anisotropic relaxation; however, the coincidence of the spectral positions is evident. This indicates that, in terms of magnetic properties, the coordination environment of the vanadium ion does not significantly change upon dissolution in the water/glycerol mixture.

**Figure 6:**
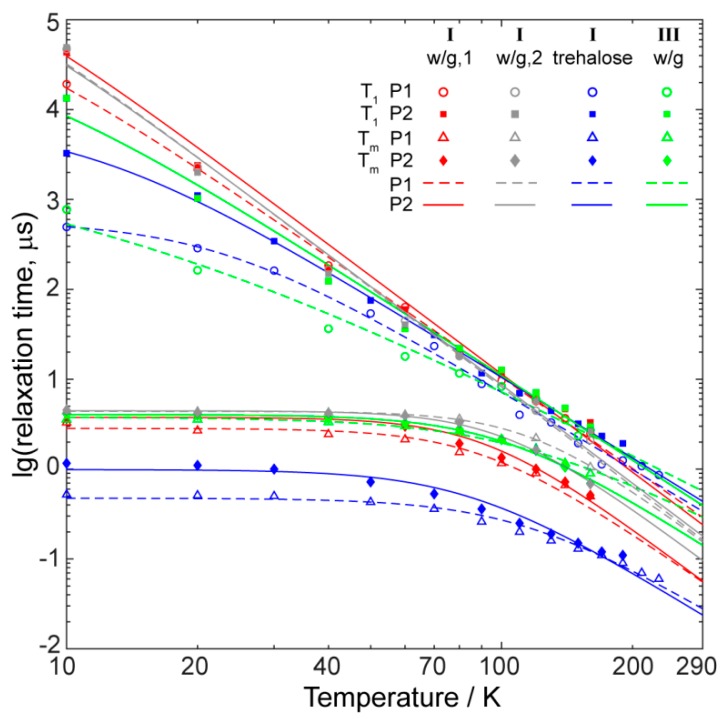
Relaxation times (*T*_1_, *T*_m_) vs. temperature for the compounds **I** and **III** in water/glycerol mixtures and in glassy trehalose. All measurements were done in two spectral positions P1 and P2 (see Figure 5a and text). Solid and dashed lines are the best fits using equations (1) and (2). *w*/g,1 and *w*/g,2 correspond to the concentrations ~10 mM and ~0.3 mM, respectively. The error of relaxation measurement does not exceed 20% at any temperature.

Figure 6 shows the dependence of *T*_1_ and *T*_m_ on the temperature measured for compounds I and III in water/glycerol glass. In crystal state, one of them (I) has a molecular structure, while another one (III) has a polymer-chain structure. Following the work of Sessoli et al. [6], we have measured the relaxation times in two spectral positions (indicated as P1 and P2, see Figure 5a). P1 corresponds to the powder-like position at nuclear spin projection *m*_I_ = −1/2, whereas P2 refers to the single-crystal-like position at *m*_I_ = −7/2. In order to verify the impact of concentration, the compound **I** was measured at two concentrations differing by a factor of ~30 (w/g,1 corresponds to ~10 mM, and w/g,2 to ~0.3 mM).

Temperature dependences of the longitudinal relaxation time *T*_1_ for both compounds were principally similar; however, at low temperature, the absolute values of relaxation differed by more than an order of magnitude. Since the *T*_1_ values for **I** having molecular structure were higher, we assumed that in the case of **III** polymeric chains did not fall apart completely, and local dipolar interactions between neighboring vanadium ions were stronger.

Both *T*_1_ and *T*_m_ values at low temperature and for each of the two compounds have longer values at side position P2 of the spectrum compared to the central one P1, in agreement with previous observations [6]. At high temperatures, in some cases, they became close to each other and coincided within experimental accuracy.

The temperature dependence of *T*_m_ for both compounds had a plateau at low temperatures and began to deviate from it at *T* ~ 50 K. Therefore, we assumed that the shortening of *T*_m_ at *T* > 50 K was primarily caused by the acceleration of the longitudinal relaxation at higher temperatures.

The obvious weakness of using the water/glycerol glass was that it melted at relatively low temperatures of ~170 K, and *T*_m_ measurements at higher temperatures became impossible. At the same time, relaxation at ambient temperatures was the most interesting for prospective applications of vanadium-based quantum qubits. In an attempt to overcome this temperature limitation of water/glycerol glass, we introduced an alternative approach. Since all studied compounds were well soluble in water, we prepared the concentrated water solution of disaccharide trehalose, dissolved the compounds in this solution, and then obtained the trehalose glass by lyophilization and evacuation (see Materials and Methods). The same approach was previously implemented in biological applications of EPR for the immobilization of double-stranded DNAs [17]. The glassy trehalose powder with incorporated vanadium blocks can, in principle, be studied by pulse EPR at all temperatures from liquid helium to the ambient ones. However, strong nuclear modulation arising from protons of trehalose complicates a lot the *T*_m_ measurement in P2 at nearly room temperatures. Furthermore, as is shown in Figure 6, the obtained relaxation times (both *T*_1_ and *T*_m_) in trehalose were systematically shorter compared to those in water/glycerol glass for the same compound. This clearly means that the distribution of vanadium centers in glassy trehalose was not homogeneous, leading to relatively high local concentrations of spin centers and shortened relaxation times. At *T* ~ 160 K, the *T*_1_ values in trehalose approached those in water/glycerol glass, but *T*_m_ values still remained much shorter. Thus, the *T*_m_ data in trehalose at room temperature provides strongly underestimated *T*_m_ values compared to those expected for well magnetically-isolated vanadium blocks.

Therefore, rather than referring to *T*_m_ values in trehalose, it would be more useful to extrapolate the data in water/glycerol mixture theoretically from ~160 to 300 K to have at least an estimate of *T*_m_. As was mentioned above, the *T*_m_(*T*) dependence indicates that shortening at *T* > 50 K was induced by the acceleration of longitudinal relaxation. Therefore, first, we performed modeling of *T*_1_(*T*) dependence using the equation [6]:

(1) $$\frac{1}{T_{1}}=A\cdot T+B\cdot T^{n}+C,$$

where *A*, *B*, *C,* and *n* are variable parameters. The first term in the right part of this equation accounts for the direct process, whereas the second one describes the Raman-like relaxation process [6]. It was not necessary to include contributions of phonon bottleneck and Orbach processes in the simulations, therefore, these terms were omitted. The obtained parameters of the fitting are given in Table S4, where the most important parameter *n* of the Raman-like process was found to be close to *n*=3, similar to previous studies of relaxation in vanadium(IV) qubits [6]. Next, we phenomenologically assume that:

(2) $$\frac{1}{T_{m}}=\frac{1}{T_{m0}}+\frac{k}{T_{1}},$$

with *T*_m0_ being the value of *T*_m_ at a low temperature limit, and *k* being the phenomenological variable parameter. Thus, we fit the *T*_1_(*T*) and *T*_m_(*T*) dependences in water/glycerol at *T* = 10–160 K, and then extrapolate the dependence using the obtained parameters up to the ambient temperatures. The results of extrapolation are shown in Figure 6, and all calculation parameters are given in Supporting Information.

In order to validate the above extrapolation approach, we also applied it to the (otherwise unsuccessful) data in trehalose. Namely, we cut data at *T* = 160 K for both *T*_1_ and *T*_m_ dependences, simulated them both using equations (1) and (2), and then extrapolated up to the room temperatures. Figure S3 shows that this extrapolation yielded reasonable agreement with factual experimental data at *T* > 160 K, therefore, this phenomenological extrapolation seemed to be well suitable for estimation of *T*_1_ and *T*_m_ at room temperature. In the future, we have to validate this approach in more detail by synthesizing a set of magnetically-diluted solid compounds in order to make it quantitative.

Meanwhile, we noticed that the decoherence times *T*_m_ of the compounds **I** and **III** at room temperature were lower than the record 1 µs value for vanadyls [6]. Still, the estimated (maximum) values *T*_m_ ~ 0.1 µs for **I** and *T*_m_ ~ 0.3 µs for **III** were not excessively small and, essentially, were comparable to those obtained in other vanadyls [5,6,11]. At this intermediate stage of research, we do not aim for a detailed analysis of room-temperature *T*_m_ vs. compound and sample preparations (Figure 6). We assume that the accuracy of extrapolation would not allow definite conclusions in this case (see Supporting Information); however, this can be developed furthermore in the course of this project.

Although all relaxation data were obtained for the representative compounds **I** and **III**, we believe that the closeness of the structure and CW EPR parameters between **I** and **II**, and between **III** and **IV**, respectively, strongly suggests that similar results would be found for compounds **II** and **IV** as well. Despite the differences observed at low temperatures (Figure 6), the *T*_1_ and *T*_m_ values of all compounds near room temperature were reasonably close for low-concentrated samples.

The vibrations (phonons) have been called a major factor controlling the electron relaxation of vanadyls at room temperatures [8]. Speaking of octahedral VO_6_ complexes, one could, in principle, anticipate some manifestations of the dynamic Jahn–Teller effect [21,22,23], which are generally unwelcome for long decoherence. However, the influence of such vibrations, if present, can be further investigated and, perhaps, adjusted by the ligand environment. Our present work shows that the room-temperature relaxation properties of VO_6_ units were not decisively worse than those of other studied vanadyl units, and, therefore have to be researched in more detail in the future.

## 3. Materials and Methods

New compounds (**III** and **IV**) were synthesized in air using deionized water as a solvent. Starting reagents were VOSO_4_·3H_2_O (>99%), cyclobutane-1,1-dicarboxylic acid (H_2_cbdc, 99%, “Acros Organics”), NaOH (>99%), Ba(NO_3_)_2_ (>98%), Y(NO_3_)_3_·5H_2_O (99.9%), Lu(NO_3_)_3_·6H_2_O (99.9%).

Synthesis of [NaLn(VO)_2_(cbdc)_4_(H_2_O)_10_]_n_ (**III**: Ln = Y, **IV**: Ln = Lu): VOSO_4_·3H_2_O (0.100 g, 0.46 mmol) was dissolved in H_2_O (15 mL), then Ba(NO_3_)_2_ (0.120 g, 0.46 mmol) was added, and the reaction mixture was stirred for 20 min at 40 °C. The solution of Na_2_cbdc obtained by neutralization of H_2_cbdc (0.133 g, 0.92 mmol) and NaOH (0.074 g, 1.84 mmol) in H_2_O (10 mL) was added to the reaction mixture and the stirring was continued. After 10 min Y(NO_3_)_3_·5H_2_O (0.112 g, 0.31 mmol) or Lu(NO_3_)_3_·6H_2_O (0.144 g, 0.31 mmol) was added. The reaction mixture was stirred for 10 min more and left to stand for 1 h, then BaSO_4_ precipitate was removed by filtration. Blue solution (25 mL) was left to evaporate slowly in air at 22 °C. Blue crystals suitable for X-ray diffraction precipitated in 2 months. The crystals were separated from the mother liquor by filtration, washed with H_2_O (*t* = 22 °C) and air-dried at 22 °C.

The yield of **III** was 0.104 g (45.5% based on VOSO_4_·3H_2_O). Anal. Calc for C_24_H_44_NaO_28_V_2_Y (%): C, 28.99; H, 4.46. Found (%): C, 28.83; H, 4.48. IR (ATR), ν/cm^–1^: 3642 v.w, 3357 br.m, 3243 m, 3000 m, 2957 m, 1633 m, 1582 s, 1556 s, 1443 m, 1431 m, 1391 s, 1349 s, 1254 m, 1242 m, 1230 m, 1196 w, 1123 m, 1061 w, 1012 w, 1000 w, 968 s, 952 s, 924 m, 876 w, 843 w, 807 w, 773 m, 762 m, 725 s, 650 s, 611 s, 561 s, 532 s, 471 s, 451 s, 442 s, 420 s.

The yield of **IV** was 0.083 (33.5% based on VOSO_4_·3H_2_O). Anal. Calc for C_24_H_44_NaLuO_28_V_2_ (%): C, 26.68; H, 4.10. Found (%): C, 26.56; H, 4.12. IR (ATR), ν/cm^–1^: 3640 v.w, 3360 br. m, 3230 m, 3000 w, 2956 w, 1632 m, 1582 s, 1555 s, 1443 m, 1431 m, 1391 s, 1349 s, 1254 m, 1242 m, 1230 m, 1195 w, 1162 w, 1123 m, 1063 w, 1012 w, 1000 w, 968 s, 953 s, 924 m, 876 w, 844 w, 807 w, 773 m, 762 m, 725 s, 654 s, 562 s, 533 s, s, 469 s, 445 s, 439 s, 421 s, 407 s.

Infrared spectra of the complexes **III** and **IV** were recorded in the frequency range from 4000 to 400 cm^−1^ on a Perkin-Elmer Spectrum 65 Fourier transform infrared spectrometer equipped with Quest ATR Accessory (Specac) accessory. Elemental analysis of the synthesized compounds was carried out on a EuroEA 3000 CHNS analyzer (EuroVector, S.p.A.). The purity of compound samples was approved by PXRD. The powder patterns were measured on a Bruker D8 Advance diffractometer with LynxEye detector in Bragg-Brentano geometry, with the sample dispersed thinly on a zero-background Si sample holder, λ(CuKα) = 1.54060 Å, θ/θ scan with variable slits (irradiated length 20 mm) from 5° to 41° 2θ, stepsize 0.02°.

EPR measurements were done using a Bruker Elexsys E580 pulse/CW EPR spectrometer (Bruker Biospin, Rheinstetten, Germany) at X-band (9.7 GHz). The spectrometer possessed a cryostat and Oxford Instruments temperature control system allowing measurements within a range of 4 to 300 K. For CW measurements, polycrystalline powder samples were prepared, placed into quartz capillary tubes, and measured as they were. Further simulations we performed using EasySpin software for MatLab [24].

Pulse EPR measurements were performed for 2 representative compounds (**I** and **III**) in a frozen solution of water/glycerol or in glassy trehalose. In the first case, the compounds were dissolved in a water-glycerol mixture, shock-frozen and measured vs. temperature starting from the lowest limit (10 K). In the second case, the compounds were dissolved in a water-trehalose mixture, then shock-frozen in liquid nitrogen and lyophilized at low pressure (~10^−2^ Torr) to gain a powdered sample (of glassy trehalose), and then placed into a sample tube. This procedure followed our previous work [17]. Note that in both of these cases, we aimed at diamagnetic dilution of the original solids containing vanadium-based building blocks, but still, we used rather concentrated samples from the point of view of common relaxation measurements in frozen solutions. The approximate concentrations in both water/glycerol and trehalose varied between 10 and 50 mM. Such concentrations corresponded to a roughly ~3 nm average distance between paramagnetic centers in frozen solution. In one of the previous work [3] in solution, much lower concentrations were used (~1 mM), resulting in a separation between spin centers of about 11 nm. At the same time, some other experiments in diamagnetically diluted solids used a noticeably higher density of spins with a spin-spin separation of about 1 nm [6]. From general considerations, it is clear that any applications of qubits would require their spatial density to be rather high. Therefore, for the diagnostics of the relaxation properties of vanadium centers in the present work, we did not aim at extreme degrees of dilution. Moreover, the room-temperature decoherence times should not be strongly dependent on the spin concentration within the above-mentioned limits. For verification, we also prepared and investigated the sample of **I** in water/glycerol in a concentration ≈0.3 mM.

For transverse relaxation measurement, we employed a 2-pulse Hahn echo sequence (π/2 - τ - π - τ - echo), where interpulse delay τ was incremented, and pulse lengths used were 10–14 ns for π/2 and 20–28 ns for π pulses. For longitudinal relaxation time measurements, we used saturation-recovery sequence with pulse-train (PT) instead of the low-power saturating pulse: PT – T_0_ - π/2 - τ - π - τ - echo, where T_0_ was incremented and τ was fixed, and PT = [π - τ_0_ -]_n_ with n~4–5 and τ_0_ being approximately the time where inverted echo crosses zero level. The pulse lengths were the same as those used for transverse relaxation measurements.

The X-ray diffraction data set for compound **III** was collected on a Bruker SMART APEX II diffractometer equipped with a CCD detector (Mo-K_α_, λ = 0.71073 Å, graphite monochromator) [25]. For both compounds, semiempirical absorption corrections were applied [26]. The structure was solved by direct methods and refined by the full-matrix least-squares with anisotropic displacement parameters using the SHELX-2014 [27] and Olex2 [28] program packages. The hydrogen atoms were positioned geometrically and refined using the riding model. The crystallographic parameters and the structure refinement statistics for complex **III** at *T* = 120 K were as follows: C_24_H_44_NaO_28_V_2_Y, *M*w = 994.37 g·mol^−1^, blue prismatic crystals, space group *C*2/*c*, *a* = 9.077(3), *b* = 24.606(8), *c* = 17.017(5) Å, *β* = 104.752(7)°, *V* = 3676(2) Å^3^, *Z* = 4, *ρ*_calc_ = 1.797 g cm^–3^, *μ* = 2.18 mm^–1^, 2.46 ≤ *θ* ≤ 30.17°, 17577 measured reflections, 2815 reflections with *I* > 2σ(*I*), *R*_int_ = 0.0936, *GooF* = 1.088, *R*_1_(*I* > 2σ(*I*)) = 0.0682, *wR*_2_(*I* > 2σ(*I*)) = 0.1767, *R*_1_(all data) = 0.0876, *wR*_2_(all data) = 0.1931, *T*_min_/_max_ = 0.4194/0.7461. The structural data for compound **III** were deposited with the Cambridge Crystallographic Data Centre (CCDC 1964259; http://www.ccdc.cam.ac.uk/products/csd/request/).

## 4. Conclusions

Long electron spin decoherence times of vanadium(IV) complexes, especially at room temperatures, have attracted significant attention during recent years. Several ways of molecular design have been employed thus far, aiming at the removal of nuclear spin-carrying atoms from the vicinity of the vanadium center, and at adjusting the vibrational modes influencing relaxation. The *T*_m_ values higher than 1 µs at room temperature still have to be achieved, and the research in this direction still continues in several groups.

In this work, we have mapped the magnetic properties of several vanadium(IV) complexes with lanthanides for potential future directions in this field. We assume that, at some point, vanadium-based qubits can be combined with other functionalities provided by lanthanides, e.g., single-molecule magnetism or luminescence. As the first step in that direction, we have studied the magnetic properties of V-Ln heterometallic complexes. It has been found that the *T*_m_ values in complexes of vanadium(IV) with diamagnetic lanthanides are generally comparable to those exhibited by other vanadium-based qubits specially designed for this purpose [3,4,5,6,7,8,9,15]. First of all, this owes to the nuclear spin-free first coordination sphere of the VO_6_ moiety. We also did not observe the strongly detrimental influence of the dynamic Jahn–Teller effect at room temperature. In order to investigate relaxation properties, we dissolved the initial compounds in a water/glycerol mixture, performed pulse EPR measurements within *T* = 10–160 K in the glassy state, and then theoretically extrapolated the data up to room temperature, validating such approach by auxiliary experiments in glassy trehalose. Altogether, this approach provides a useful methodology for facile estimation of room-temperature *T*_m_ values in vanadium-based qubits. The study of other vanadium complexes of lanthanides, including those with paramagnetic lanthanide ions, is underway in our laboratories.

## Figures and Tables

**Table 1:** Primary spectroscopic parameters (g- and A-tensors) used in simulations shown in Figure 4 for studied vanadium(IV) complexes in the solid-state. The accuracy of g-tensor is ~0.001, for A-tensor ~0.5 MHz. The subscripts ⊥ and ‖ refer to the perpendicular and parallel components of the corresponding tensors.

Compound	[g_⊥_; g_‖_]	[A_⊥_; A_‖_]/MHz
**I**	[1.979; 1.938]	[187; 529]
**II**	[1.978; 1.937]	[189; 525]
**III**	[1.977; 1.941]	[157; 520]
**IV**	[1.977; 1.943]	[151; 512]

## Supplementary Materials

The following are available. Figure S1 (PXRD data), Tables S1–S3 (structural data), Figure S2 (magnetic data for I, II), Figure S3 (Tm extrapolation data), Table S4 (simulation parameters).
