# Supplementary material for: Mapping Magnetic Properties and Relaxation in Vanadium(IV) Complexes with Lanthanides by Electron Paramagnetic Resonance
Source: Molecules. 2019 Dec 14;24(24):4582. doi: 10.3390/molecules24244582 (PMC6943608; doi:10.3390/molecules24244582)
Supplement: Supplementary file 1 [file molecules-24-04582-s001.pdf]

SUPPORTING INFORMATION

Mapping Magnetic Properties and Relaxation in Vanadium(IV) Complexes with Lanthanides by Electron Paramagnetic Resonance

Ivan V. Kurganskii [1,4], Evgeniya S. Bazhina [2], Alexander A. Korlyukov [3], Konstantin A. Babeshkin[2], Nikolay N. Efimov [2], Mikhail A. Kiskin [2], Sergey L. Veber [1,4], Alexey A. Sidorov [2], Igor L. Eremenko [2,3] and Matvey V. Fedin [1,4]*

1 International Tomography Center SB RAS, 630090 Novosibirsk, Russia
2 N.S. Kurnakov Institute of General and Inorganic Chemistry of the Russian Academy of Science, 119991 Moscow, Russia
3 Nesmeyanov Institute of Organoelement Compounds of the Russian Academy of Sciences, 119991 Moscow, Russia
4 Novosibirsk State University, 630090 Novosibirsk, Russia
* Correspondence: mfedin@tomo.nsc.ru

I. Supplementary PXRD data

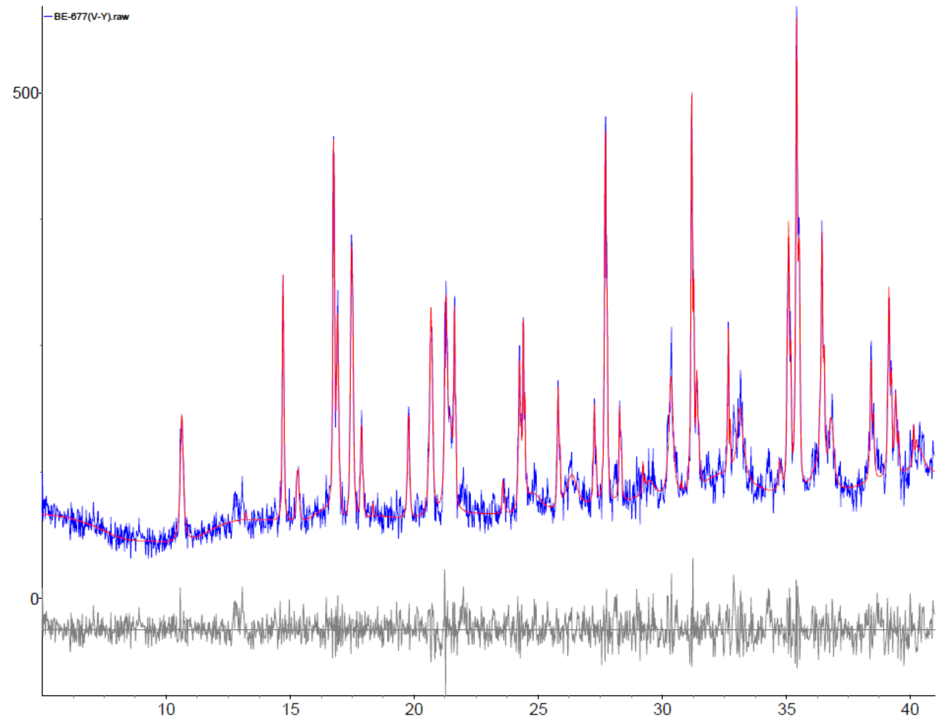

a

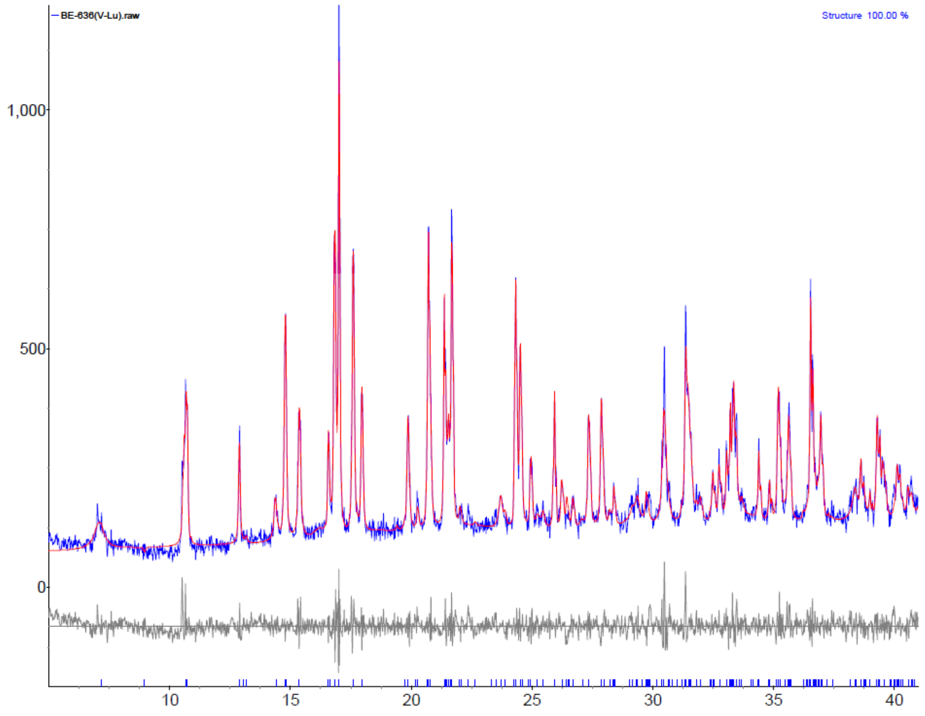

b

Fig. S1. Experimental PXRD patterns (blue line) for **III** (*a*), **IV** (*b*) and their comparison with calculated data (red line) for **III** and their difference (grey line). Measured at room temperature.

II. Supplementary structural data

Table S1. Selected bond lengths, the shortest interatomic distances *d* (Å) and angles ω (°) in complex **III**.

Bond	*d*	Bond	*d*
Y1–O2W	2.327(4)	V1–O5W	2.283(4)
Y1–O3W	2.356(4)	V1–O6	1.991(4)
Y1–O4W	2.355(4)	V1–O7	2.013(4)
Y1–O9	2.343(3)	Na1–O1W	2.437(4)
V1–O1	1.598(4)	Na1–O5W	2.407(4)
V1–O2	1.975(4)	Na1–O6	2.625(4)
V1–O3	1.966(4)		
Distance	*d*	Distance	*d*
V1···V1	6.351	V1···Y1	5.687
Angle	*ω*	Angle	*ω*
O1–V1–O2	99.63(17)	O7–V1–O5W	81.71(14)
O1–V1–O3	101.25(18)	O2–V1–O6	85.43(15)
O1–V1–O6	101.48(18)	O3–V1–O2	89.64(15)
O1–V1–O6	100.27(17)	O3–V1–O7	90.06(15)
O2–V1–O5W	78.31(14)	O6–V1–O7	87.05(14)
O3–V1–O5W	80.16(14)	O1–V1–O5W	177.53(18)
O6–V1–O5W	77.07(14)		

Table S2. Hydrogen bond parameters in structure **III**.

Fragment D–H···A	Bond length/ Å			D–H···A /°
	D–H	H···A	D···A	
O1W–H1WA···O4$^{(i)}$	0.85	2.13	2.813(5)	137.4
O2W–H2WA···O8$^{(i)}$	0.86	1.89	2.706(5)	158.3
O2W–H2WB···O7$^{(ii)}$	0.86	2.07	2.699(5)	129.6
O3W–H3WA···O1$^{(iii)}$	0.88	1.91	2.736(6)	156.7
O3W–H3WB···O4$^{(iv)}$	0.87	1.93	2.798(5)	170.7
O5W–H5WA···O8$^{(i)}$	0.85	1.91	2.728(5)	160.0
O5W–H5WB···O5$^{(ii)}$	0.85	2.21	2.758(5)	121.8

Symmetry codes: (i) –x,+y,$^{1}/_{2}$–z; (ii) 1–x,+y,$^{1}/_{2}$–z; (iii) 1–x,1–y,1–z; (iv) $^{1}/_{2}$+x,$^{1}/_{2}$+y,+z.

Table S3. Selected bond lengths *d* (Å) and angles ω (°) in complexes **II** and **III**.

**Parameter/Complex**	**II**	**III**
Bond	d	
V=O	1.585(2), 1.594(2)	1.598(4)
V–O(cbdc)	1.989(2)–2.019(2)	1.966(4)–2.013(4)
V–O(H_2O)	2.272(2), 2.407(2)	2.283(4)
Angle	ω	
V=O / V–O_{eq}	97.06(9)–101.88(10)	99.63(17)–101.48(18)
V=O / V–O_{ax}	175.32(9), 178.09(10)	177.53(18)
V–O_{eq} / V–O_{eq}	86.35(8)–90.77(8)	85.43(15)–90.06(15)
V–O_{eq} / V–O_{ax}	74.44(8)–82.30(8)	77.07(14)–81.71(14)

III. Supplementary magnetometry data

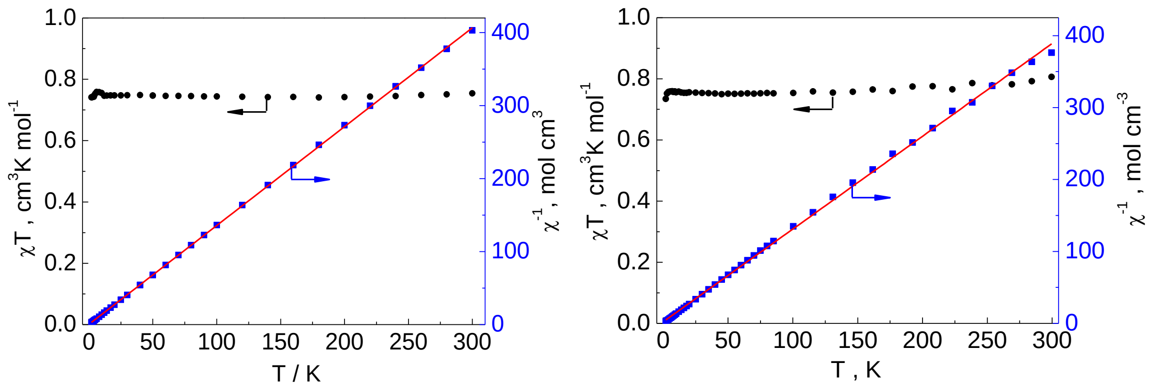

(a) (b)

Figure S2. Temperature dependences of magnetic susceptibility for **I** (a) and **II** (b). Results previously reported in Ref.[19].

IV. Supplementary EPR data

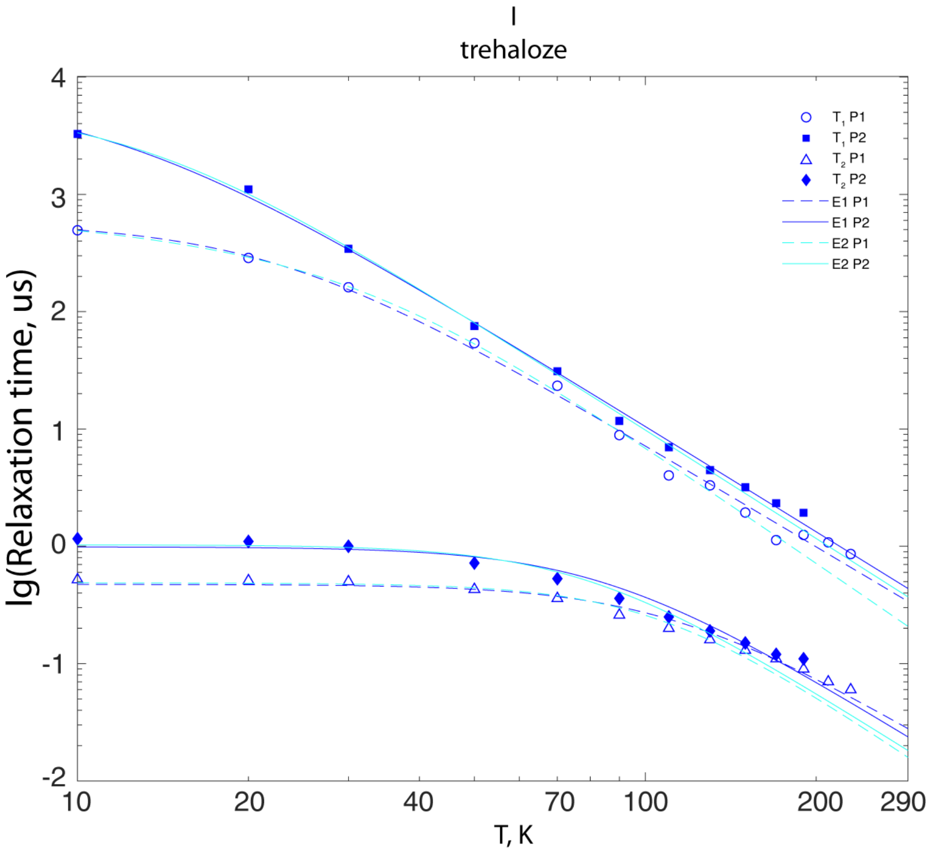

Fig. S3. Validation of high temperature extrapolation of T_1 and T_m in trehalose case. E1 stands for the first type of extrapolation – the extrapolation based on all experimental points. E2 stands for the second type of extrapolation – based on experimental points up to 150 K.

In order to validate the extrapolation approach used in the main text, we applied it also to the (otherwise unsuccessful) data in trehalose. Namely, we cut data at T=150 K for both T_1 and T_m dependences, simulated them both using equations (1) and (2) MT, and then extrapolated up to the room temperatures. Figure S3 shows that this extrapolation yielded reasonable agreement with factual experimental data at T>150 K, therefore this phenomenological extrapolation seems to be well suitable for estimation of T_1 and T_m at room temperature. The accuracy of such extrapolation can be estimated form the span of the values at 290 K, being roughly within 0.2 μs for T_m. In the future, we have to validate this approach in more detail by synthesizing a set of magnetically-diluted solid compounds, in order to make it quantitative.

Table S4 reports the simulation parameters (being also the extrapolation parameters) for the dependencies of relaxation times on temperature. The curves themselves are presented in Figures 6 of the main text and in Figure S3 of SI.

Table S4. Parameters of relaxation times vs temperature extrapolations. See eq. 3 and 4 in the main text.

T_1 in P1	n	A $(s\cdot K)^{-1}$	B $(s\cdot K^n)^{-1}$	C s^{-1}
I w/g,1	*3.68*	*0.54*	*0.01*	*0.00*
I w/g,2	*3.37*	*1.73*	*0.02*	*0.00*
I treh E1	*2.89*	*22.79*	*0.23*	*1605.70*
I treh E2	*3.34*	*83.17*	*0.03*	*1144.05*
III w/g	*2.47*	*144.98*	*1.45*	*0.00*

T_m in P1	k	T_{m0} µs
I w/g,1	*1.02*	*2.20*
I w/g,2	*5.15*	*1.42*
I treh E1	*11.43*	*0.24*
I treh E2	*12.66*	*0.25*
III w/g	*1.79*	*1.91*

T_1 in P2	n	A $(s\cdot K)^{-1}$	B $(s\cdot K^n)^{-1}$	C s^{-1}
I w/g,1	*3.64*	*0.61*	*0.01*	*0.00*
I w/g,2	*3.63*	*0.49*	*0.00*	*0.00*
I treh E1	*2.99*	*9.69*	*0.10*	*100.99*
I treh E2	*3.08*	*6.99*	*0.07*	*144.58*
III w/g	*3.13*	*5.04*	*0.05*	*0.00*

T_m in P2	k	T_{m0} µs
I w/g,1	*1.83*	*2.23*
I w/g,2	*4.17*	*1.88*
I treh E1	*17.97*	*0.50*
I treh E2	*20.05*	*0.52*
III w/g	*2.68*	*2.02*
